# Sexual health of very young adolescents in South Western Uganda: a cross-sectional assessment of sexual knowledge and behavior

**DOI:** 10.1186/s12978-018-0595-3

**Published:** 2018-08-29

**Authors:** Elizabeth Kemigisha, Katharine Bruce, Viola N. Nyakato, Gad Ndaruhutse Ruzaaza, Anna B. Ninsiima, Wendo Mlahagwa, Els Leye, Gily Coene, Kristien Michielsen

**Affiliations:** 1 Mbarara University of Science and Technology, P.O. Box 1410, Mbarara, Uganda; 2 International Centre for Reproductive Health, Faculty of Medicine and Health Sciences, Ghent University, Ghent, Belgium; 3 RHEA, Centre of Expertise on Gender, Diversity and Intersectionality, Vrije Universiteit Brussel, Brussels, Belgium

**Keywords:** Young adolescents, Sexuality, Uganda

## Abstract

**Background:**

In most Sub-Saharan African countries, little is known about young adolescents’ sexual and reproductive health (SRH). Though some efforts have been made to understand and improve SRH of older adolescents, very young adolescents (VYAs) are often overlooked, and little is known about their sexual knowledge and behaviors. The goal of this study was to describe SRH knowledge, information-seeking, and sexual behavior of VYAs in Uganda.

**Methods:**

A cross-sectional survey was administered in 33 primary schools in June and July of 2016. Trained interviewers administered surveys to adolescents ages 10–14 regarding SRH knowledge, information-seeking, sexual behavior, and relevant covariates. Continuous variables were summarized as means (SD) or medians (IQR) whereas categorical variables were summarized as proportions (percentages).

**Results:**

A total of 1096 adolescents were included in this analysis, 81.8% of which were from rural areas, with a median age of 12. Regarding sexually transmitted infections (STIs) knowledge; 95% knew HIV while 37% knew other STIs apart from HIV. Although 47% knew at least one way in which HIV is acquired only 8% knew at least four ways. Regarding contraceptive knowledge, 56% mentioned at least one modern method of preventing pregnancy (condoms, pills, intrauterine devices, implants, or injections). The majority (85%) of VYAs reported accessing SRH information in the media with 35% reporting accessing media with sexual content while 10% vs 22% consulted their father or mother respectively and 31% a school source. At least 7.6% of VYAs had ever had sexual intercourse, 90% of which were not using any protection.

**Conclusion:**

Comprehensive SRH knowledge was low among VYAs in this study. Media remains an important source of information for SRH for this age group though it may be misused as some adolescents reported accessing sexual content that may be inappropriate. A large proportion of sexually active VYAs reported sexual risky behaviors. This study highlights the need for an accurate and more comprehensive SRH education approach for VYAs in Uganda at an opportune age before the majority engage in sexual behavior.

## Plain English summary

Uganda has the second youngest population in the world, with high rates of sexually transmitted diseases and adolescent pregnancy. Access to sexual and reproductive health (SRH) information and resources is a challenge, especially for very young adolescents (VYAs) aged 10–14. The goal of this study is to assess levels of SRH knowledge, SRH information seeking and sexual behavior among VYAs. We found that most students are aware of HIV, but few have detailed knowledge of how HIV is acquired, what other STIs exist, and what methods of pregnancy prevention are available. Seven percent of VYAs were already sexually active with most of them not using any form of protection such as condoms. The majority of VYAs had been exposed to SRH information in the media (television, radio or newspapers) with at least one third of them accessing media with sexual content, while far fewer had received such information in schools or from their parents. This study highlights the importance of implementing school-based SRH education at a young age. Because the majority of VYAs are not yet sexually active, SRH interventions in this age group may be more effective than those in older adolescents in delaying sexual onset and decreasing risky sexual behavior. Furthermore, because these students are already being exposed to sexual information in the media, it is important for schools and parents to provide accurate information that helps students to reflect more critically on SRH information in media sources.

## Background

With a median age of just 15, Uganda has the second youngest population in the world [1]. Over half of the population is the under age of 18 years [2]. This youthful population presents unique challenges, especially with regard to sexual and reproductive health (SRH). Uganda’s 2016 Demographic and Health Survey (DHS) found that 24.8% of girls ages 15–19 had already begun childbearing, which was as high as 53.9% among girls age 19 [3]. Uganda has one of the ten highest HIV prevalence rates in the world at 6.2% (4.7% among males and 7.6% among females) [4, 5]. Despite great initial success in decreasing HIV prevalence and incidence throughout the 1990s and early 2000s [6], HIV/AIDS remains the leading cause of death in Uganda [7], and the majority of new infections occur among adolescent women [3]. In this young population, comprehensive access to sexual and reproductive health knowledge and services is of upmost importance.

Access to SRH information and prevention services remains a challenge in most developing countries, including Uganda. The 2016 DHS found that only 40% of Ugandan adolescents ages 15–19 years have comprehensive HIV prevention knowledge [3]. A study among girls in Ugandan primary schools found low knowledge of menstruation and menstrual hygiene [8]. Inadequate SRH knowledge creates anxiety and shame, especially at the beginning of puberty [9]. While there is little data available on SRH outcomes among VYAs in Uganda, poor reproductive health outcomes become especially evident in late adolescence. A study among university students found high non-use of contraception, inconsistent condom use and low knowledge of proper condom use, especially among females and those who had early sexual debut [10, 11]. It is possible that intervening in early adolescence could mitigate adverse outcomes in older adolescence.

Despite these challenges, there has been a lot of pushback on SRH education in Uganda from parents, teachers, and even Members of Parliament (MPs), who feel that teaching these topics in schools will erode Uganda’s culture and morality [12]. In 2016, the Ministry of Gender, Labor and Social Development banned sexuality education in schools [13]. The Ministry of Education and Sports recently released new national guidelines on sexuality education, but these guidelines are not comprehensive, they emphasize abstinence, and the future of their implementation is still unclear [14]. With no standardized SRH curriculum in schools and ongoing religious and political pushback on its relevance and appropriateness, adolescents in Uganda are left with few options to seek SRH information.

Following evidence on the proven benefits of comprehensive sexuality education in diverse settings [15], UNESCO recently released guidelines outlining recommendations for comprehensive sexuality education [16]. However, most of the existing SRH interventions in low and middle income countries, as well as most of the existing research, focus on older adolescents [15]. SRH needs of very young adolescents (VYAs), those aged 10–14 years, are often neglected in research and action [17–19] and there are many gaps in understanding SRH of young adolescents, especially in Sub-Saharan Africa (SSA) [20, 21]. This younger age group may be the ideal target for SRH interventions, as their behaviors and norms are less rigid [22]. Due to the fact that the majority of VYAs in Uganda are not yet sexually active [23], they are more susceptible to behavioral interventions than their older peers who have already started engaging in risky sexual behaviors. Bankole et al., 2007 found that 11% of Ugandan adolescents ages 12–14 are already sexually active [23], showing that SRH information is indeed relevant for this young age group, who are beginning to be exposed to SRH risks. Furthermore, research by Kemigisha et al., 2018 on Ugandan VYAs found that inequitable norms around gender and sexuality increase with age [24]. Harmful gender norms surrounding sexuality, consent and decision-making exacerbate SRH risks for boys as well as girls [25]. Interventions should target adolescents at a young age, before they have already developed harmful norms about gender and sex.

The goal of this study is to investigate the current status of sexual behavior, SRH knowledge, and information-seeking behaviors of adolescents ages 10–14 in Uganda. It aims to fill the gap in the literature regarding the SRH of VYAs in Uganda. This information should guide the development and implementation of educational SRH interventions across the country.

## Methods

### Study design and setting

This paper presents the results of a cross-sectional survey of very young adolescents in primary schools in Mbarara district of South Western Uganda. This data was collected as the baseline of a cluster randomized trial to evaluate the implementation and effectiveness of a comprehensive sexuality education program in primary schools. According to the 2015 District Education reports, Mbarara has a total of 248 primary schools with an estimated primary school enrolment of 80,924 [26].

### Study population and selection of participants

Participant eligibility criteria included being 10–14 years old and in primary level 5 or 6 of education from rural and urban schools within Mbarara district. The final sample size included 1096 adolescents from 33 schools in both urban and rural settings. A list of primary schools in Mbarara was available in district records. We eliminated schools that had no upper classes (primary 5 to 7), and schools that offer unique services, including one school for children with hearing impairments and one school for children with visual impairments. The remaining schools were entered into an Excel spreadsheet and a formula was developed to randomly select the required 33 schools for the study. A second Excel formula was used to randomly assign each of the 33 schools to the treatment or control arm. Research assistants (RAs) selected the final sample of students in each school from class registration lists using systematic sampling methods and substituting pupils who were absent on the day that data was collected.

### Data collection

The data were collected between June and July 2016. Standardized closed surveys were administered by trained RAs and were crosschecked by the onsite investigator and research monitors and cleaned for any errors. All RAs were fluent in both English and Runyankole (the local language) and surveys were administered based on each students’ preference. Responses to all questions were recorded and coded either using scales or binary yes/no questions, with the option to refrain from responding. Additionally, questions that assessed pupils’ knowledge included the option to respond with “I do not know”. Anonymity was maintained throughout the study and participants’ responses were deidentified.

### Definitions for outcome variables

*Sexual behavior* was measured starting from a screening question: “have you ever been to a secret place alone with a friend of the opposite sex?” If the answer to this question was yes, further questions were asked regarding sexual experiences. These included “Did you ever kiss on the lips?”, “Did you or your friend ever touch your private parts?”, “Have you ever had sex?” “How old were you when you first had sex?”, “What method did you use to prevent HIV/AIDS or STIs?” and “Were you willing to have sex the first time you did?”

*Sexual and reproductive health knowledge* was measured based on knowledge of puberty, HIV/STIs and pregnancy prevention. This included knowledge on how HIV/AIDS can be acquired, types of common Sexually Transmitted Infections, knowledge of pubertal changes in boys (such as having beards, deep voice etc.) and in girls (such as increase in breast size, having pubic or axillary hair etc.), and knowledge about ways to prevent pregnancy (such as abstinence, condom use or any other type of contraceptive).

*Sources of Information on SRH* were assessed. We asked students if they had sought SRH information from their mother, father, a school source, a community health facility and/or mass media sources. Media was further categorized into seeking information on SRH topics or sexual content.

Data was collected on socio-demographic variables such as age in years, pubertal stage, gender, religion, socio-economic status (assessed as a sum of the score for best water source, housing possessions and pupil possessions such as shoes or school uniform pairs), whether the school was located in rural or urban area and whether one, both or neither parents were alive.

### Statistical analyses

Data was double entered into Epi-data 3.1 (EpiData, Odense, Denmark). The data utilized in this analysis represent the baseline data of a cluster randomized control trial evaluating the effectiveness of a Comprehensive Sexuality Education intervention. Based on an intra-class correlation calculated in a pilot test among 105 VYAs and an estimated drop-out rate of 20%, we determined that a minimum sample size of 1100 students was needed to measure a 10% score difference in outcomes between baseline and endline among the intervention vs control groups with a power of 90%. Data analysis was done using Stata® (College Station, Texas, USA). Continuous variables were summarized as means (SD) or medians (IQR) whereas categorical variables were summarized as proportions (percentages). In addition, a bivariate analysis among categorical variables was made using chi square. All analyses were stratified by gender. A *p*-value of less than 0.05 was considered a significant association.

## Results

### Description of participants

A total of 1096 pupils were interviewed between June and July 2016. The median age was 12 (IQR 11, 13) years and 58% were female. Over 80% of the study population were from rural areas and over 80% of the pupils had both parents alive. Twenty-nine percent were ranked as having a high socio-economic score, 47% ranked medium and 24% ranked low (Table 1). The majority of pupils had achieved some form of puberty, with 90% beyond (self-assessed) tanner stage 1 (i.e. beyond normal size of penis of 3 cm/testicular volume of 1.5 ml in boys or lack of glandular tissue or no areola enlargement in girls and no pubic hair in both) and about half were at tanner stage 2 (i.e. showing early signs of breast, bud development or testicular enlargement with pubic hair). About 26% of the girls had started menstruation and 23% of the boys mentioned they had experienced wet dreams.

**Table 1:** Socio-demographic characteristics of the participants by gender

	Overall, n (%)	Male n (%)	Female n (%)	*p*-value
Gender				
Boys	460 (42.0)			
Girls	636 (58.0)			
Age in years, median (IQR)	12 (11,13)			
Pubertal age (Tanner 1–5)				
Tanner 1	130 (11.9)	49 (10.6)	81 (12.7)	1
Tanner 2	379 (34.6)	168 (36.5)	211 (33.2)	
Tanner 3	487 (44.4)	216 (47.1)	271 (42.6)	
Tanner 4 or 5	100 (9.1)	27 (5.8)	73 (11.5)	0.007
Ever experienced menstruation (female only)	155 (25.9)	–	155 (25.9)	NA
Ever experience wet dreams (male only)	101 (22.5)	101 (22.5)	–	NA
Education level				
= Primary 5 only	563 (51.4)	240 (52.2)	323 (50.8)	
= Primary 6 only	533 (48.6)	220 (47.8)	313 (49.2)	0.650
Socio economic status^a^				
Low	315 (29.0)	128 (28.2)	187 (29.6)	
Medium	511 (47.1)	215 (47.4)	296 (46.8)	
High	260 (23.9)	111 (24.4)	149 (23.6)	0.871
Religion				
Catholic	405 (37.1)	165 (36.0)	240 (37.8)	
Anglican	507 (46.4)	215 (46.9)	292 (46.0)	
Moslem	101 (9.2)	48 (10.5)	53 (8.4)	
Other	80 (7.3)	30 (6.6)	50 (7.8)	0.527
Location of school				
Rural	896 (81.8)	368 (80)	529 (83.2)	
Urban	199 (18.2)	92 (20)	107 (16.8)	0.178
Number of parents alive				
Both alive	905 (82.6)	378 (82.2)	527 (82.9)	
None/Single parent	124 (17.4)	82 (17.8)	109 (17.1)	0.767

^a^Social economic status assessed as a sum of the score for best water source, housing possessions and pupil possessions such as shoes or school uniform pairs with a possible score range of 1–25, median of 8.1. Was categorized as Low if scores were 1–5, medium for scores 6–10 and high if scores 11 till maximum score

#### Sexual health knowledge

Regarding knowledge about puberty, 27.9% vs 12.3% of the respondents knew at least 3 of 6 physical changes in puberty in boys vs 4 of 7 changes in girls respectively. A majority of respondents (95%) knew HIV is an STI and 37% knew other STIs apart from HIV. Although 47% knew at least one way in which HIV may be acquired, only 8% knew at least 4 methods by which HIV is acquired. Regarding contraceptive knowledge, 59% mentioned abstinence, 56% mentioned any of the more effective methods of preventing pregnancy (condoms, hormonal or surgical methods), and 3% knew natural methods for contraception. More boys than girls knew of any effective ways to prevent pregnancy and the difference was significant (Table 2).

**Table 2:** Level of knowledge on Sexual and Reproductive Health by gender among Very Young Adolescents in South Western Uganda

Characteristic	Total	Male	Female	Chi	*p* value
Knowledge on pubertal signs					
Pubertal signs in boys (at least 3/6)	305 (27.9)	158 (34.4)	147(23.2)	16.58	< 0.001
Pubertal signs in girls (at least 4/7)	134 (12.3)	56 (12.2)	78 (12.4)	0.09	< 0.926
Knowledge on STIS					
Knows HIV as an STI	1037 (95.1)	441 (96.5)	596 (94.2)	3.15	0.076
Knows any STI apart from HIV	402 (36.7)	183 (39.8)	219 (34.4)	3.29	0.07
Knowledge of HIV transmission					
Knows at least 1 way of HIV acquisition	516 (47.1)	218 (47.4)	298 (46.9)	0.03	0.861
Knows 4 ways of HIV acquisition	86 (7.9)	42 (9.1)	44 (6.9)	1.81	0.179
Knows a person who looks healthy can still have HIV	710 (65.3)	350 (76.8)	360 (57.1)	54.05	< 0.001
Knowledge on pregnancy prevention					
Knows abstinence	627 (59.0%)	264 (58.9)	363 (59.1)	0.004	0.95
Knows natural methods	33 (3.1%)	21 (4.7)	12 (2.0)	6.43	0.01
Knows more effective methods	592 (55.8%)	297 (66.4)	295 (48.0)	35.50	< 0.001

### Sources of SRH information

A total of 85% of VYAs reported accessing SRH information through radio or television media. In addition 35.5% had access to media with sexual content via mobile device/television, and 44.5% accessed sexual content in print media (newspapers and magazines). More boys than girls watched visual media with sexual content. A total of 31.7% reported seeking SRH information from a source at school. Only 22.3% and 9.9% had discussions about love or sex with their mother or father respectively. Pupils were more likely to communicate with a parent of their same gender, meaning more boys communicated with fathers and more girls communicated with mothers (Table 3).

**Table 3:** Sources of information on Sexual and Reproductive Health for Very Young Adolescents in SW Uganda

Characteristic	Total	Male	Female	Chi	*p* value
Ever sought information on SRH					
Father	82 (9.9)	44 (11.5)	38 (7.2)	4.97	0.03
Mother	222 (22.3)	58 (13.9)	164 (28.4)	29.39	< 0.001
School source (of 791 who were aware of services)	251 (31.7)	57 (19.9)	194 (38.7)	28.80	< 0.001
Community health facility	61 (5.6)	29 (6.3)	32 (5.0)	1.09	0.58
Any mass media with educative SRH topics	923 (85.2)	396 (87.2)	527 (83.7)	2.66	0.10
Ever watched a movie or show with sexual content	389 (35.5)	191 (41.5)	198 (31.2)	12.45	< 0.001
Ever read a newspaper or magazine describing sexual content	487 (44.5)	220 (47.9)	267 (42.1)	3.73	0.05

### Sexual behaviors

A total of 15.1% respondents had ever been in a secluded place with a girlfriend or boyfriend; 3.1% had kissed, 10.5% had either touched or been touched by a boyfriend or girlfriend in the genital area, and 7.6% had had sexual intercourse. Overall, more boys than girls reported having experienced any of these forms of sexual behavior. Of those who were sexually active, only 9.6% used condoms the first time they had sex. Of those who were not sexually active, 11.3% reported they felt pressured to have sex, of which 6.1% were boys and 3.9% were girls (Table 4).

**Table 4:** Description of sexual behavior of Very Young Adolescents by gender

Characteristic	Total	Male	Female	chi	*p* value
Ever went in seclusion with friend of opposite sex	165 (15.1)	101 (22.0)	64 (10.1)	29.29	< 0.001
Have (been) kissed	34 (3.1)	26 (5.7)	8 (1.3)	4.20	0.04
Have (been) fondled	115 (10.5)	80 (17.4)	35 (5.5)	11.15	< 0.001
Had penetrative sex	83 (7.6)	67 (14.6)	16 (2.5)	26.78	< 0.001
Used condoms on the last sexual experience (among sexually active only)	8 (9.6)	7 (10.4)	1 (6.7)	0.26	0.61
Have no sexual experience but are under pressure to have it	114 (10.4)	69 (15.0)	45 (7.1)	26.00	< 0.001

## Discussion

This study describes the sexual behavior, sexual health knowledge, and information seeking behavior of VYAs in Uganda, in an effort to inform interventions related to adolescent SRH. We found that 7% of adolescents were sexually active, and that a majority of these were not using condoms. Detailed SRH knowledge on HIV transmission, types of STIs and contraception was low. Furthermore, media was the most utilized source for SRH information, while school and parental sources were underutilized.

In Uganda, there is a lot of pushback against SRH education from many key stakeholders, including political, religious and cultural leaders, who feel that these topics are inappropriate for children and should not be taught in schools [12]. There are no clear guidelines on what, when, and in what setting SRH information should be disseminated to students [27]. However, this study shows that regardless of whether these topics are taught in schools, adolescents receive information on sex and relationships at a young age. We found that the majority (85.2%) of VYAs had been exposed to media on SRH topics with over one third accessing media with sexual content. More boys than girls had seen a movie or show with sexual content. However, far fewer adolescents received this information in school or from their parents.

Much of the SRH information in the media that adolescents are exposed to may be inaccurate or misleading [28, 29]. Although some public health institutions in Uganda use media to provide adolescents with accurate and relevant SRH information [30, 31], it is hard for adolescents to determine which sources are accurate and which are not [28, 29]. Moreover, media with sexual content has been associated with early sexual debut among adolescents in Africa [32]. Findings from a qualitative study in Uganda indicate use of media with sexual content is perceived to contribute to persistence of inequitable norms, especially in depicting women as submissive and men being sex driven [33]. In another study in a subset of these VYAs, we found inequitable sexual socialization tendencies beginning to emerge in early adolescence [25]. Both of these studies highlighted the role that media can play in shaping VYAs ideas about sex and relationships including norms that can be harmful to both boys and girls.

Schools and parents could serve as valuable resources for adolescents in mitigating potential harm of misleading media sources. One study in Uganda found that parents were in fact effective in mediating the effects of inappropriate media use among their adolescent children [34]. Furthermore, the National Sexuality Education Framework released by the Ugandan Ministry of Education and Sports includes modules on analyzing SRH information in the media [14]. Though this framework has not yet been implemented, it may enable schools to help students identify which information sources are accurate and credible, and which sources are not.

While the majority (95.1%) of VYAs were aware of HIV, few were aware of the four most common ways that HIV is transmitted and over a third said that it is not possible for a person who looks healthy to have HIV. Similar findings about SRH knowledge were earlier described by Bankole et al., 2007 [23]. This shows that despite the extremely high level of HIV awareness, adolescents had inadequate in-depth knowledge on HIV transmission. Furthermore, only one third knew any STI other than HIV. This implies that they may not be equipped with sufficient information to protect themselves from acquiring HIV and other STIs. Knowledge of contraceptive methods was moderate, with just over half mentioning abstinence or modern methods. Inadequate contraceptive knowledge may lead to poor utilization of these methods. This is supported by a previous study among sexually active adolescents that found that contraceptive knowledge and uptake has been significantly lower in younger women compared to older women [35, 36]. Clearly there are significant gaps in the information that young people are receiving about SRH. It is important that VYAs are aware of the health risks involved in sexual activity beyond HIV.

In this study, 7.6% of VYAs were sexually active, which is lower than previous findings among the same age group in Sub-Saharan Africa of 11–15% [23, 37]. More boys than girls reported being sexually active, which may relate to underreporting among Ugandan girls, who are often less comfortable and more stigmatized for being sexually active before marriage [25] . However, it was noted in this study that the majority of sexually active young adolescents did not use condoms, which puts young adolescents at risk of STIs, HIV and early pregnancy. These findings are supported with an established association that early sexual debut is a risk factor for risky sexual practices in Uganda [38, 39]. Furthermore, recent evidence shows that adolescents especially those of better social economic background globally experience puberty at a younger age [10, 40, 41]. Simultaneously, they are choosing to get married later than previous generations [9]. Attainment of biological maturity much earlier than socioeconomic independence and marriage imposes a wider biosocial gap among adolescents that may expose them to sexual and reproductive health risks prior marriage [42].

Furthermore, because the majority of these students are still not sexually active, this is an opportune age to implement behavioral SRH interventions. A plethora of studies in low and middle income countries have shown that comprehensive SRH education is associated with delayed sexual onset [15, 43], but these programs can only delay sexual onset if they are implemented before target students are sexually active. Additionally, about 96% of Ugandan children attend some primary school, while only 34% go on to secondary education [44]. If SRH interventions only target secondary school students, the majority of Ugandan adolescents will miss out on this vital information. By targeting VYAs in primary school, SRH interventions can reach the majority of Ugandan adolescents with accurate information before they become sexually active. This study highlights the vital need for comprehensive, school-based SRH education for VYAs that will enable young people make informed decisions about their sexual health.

### Study limitations

Sexual behavior was self-reported and this being a sensitive subject, under reporting could have occurred among some participants. During data collection, we ensured privacy and tried as much as possible to have interviewers of the same sex as respondents conduct the interviews in an effort to limit reporting bias, a form of information bias. While dichotomizing certain variables was helpful in demonstrating statistical results, this may have led to a loss of relevant data. Furthermore, because this was a cross sectional survey, we could not determine the direction of causality. Finally, due to cultural and legal restrictions around homosexuality in Uganda, we were only able to ask students about heterosexual behavior, and did not ask about homosexual activity.

## Conclusion

Very young adolescents in Uganda face great challenges in SRH, having low SRH knowledge, early sexual debut and risky sexual practices. SRH education should start during early adolescence, before the majority of these students are sexually active and while most of them are still in school. Comprehensive, accurate, school-based SRH education is crucial. Further research should be conducted and used by policy makers in the development of nation-wide policies regarding SRH education for VYAs.
